# Bis(dicyanamido-κ*N* ^1^)bis­[2-(2-hydroxy­ethyl)pyridine-κ^2^ *N*,*O*]nickel(II)

**DOI:** 10.1107/S1600536809043359

**Published:** 2009-11-07

**Authors:** Ling-Qian Kong, Xiu-Ping Ju, Da-Cheng Li

**Affiliations:** a Dongchang College of Liaocheng University, Shandong 252059, People’s Republic of China; b College of Chemistry and Chemical Engineering, Liaocheng University, Shandong 252059, People’s Republic of China

## Abstract

In the title complex, [Ni{N(CN)_2_}_2_(C_7_H_9_NO)_2_], the Ni^II^ ion (site symmetry 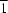) adopts a distorted *trans*-NiO_2_N_4_ octa­hedral geometry. In the crystal, inter­molecular O—H⋯N hydrogen bonds link the mol­ecules, forming a chain along the *c* axis.

## Related literature

For related structures, see: Boskovic *et al.* (2002 ▶); Sanudo *et al.* (2003 ▶).
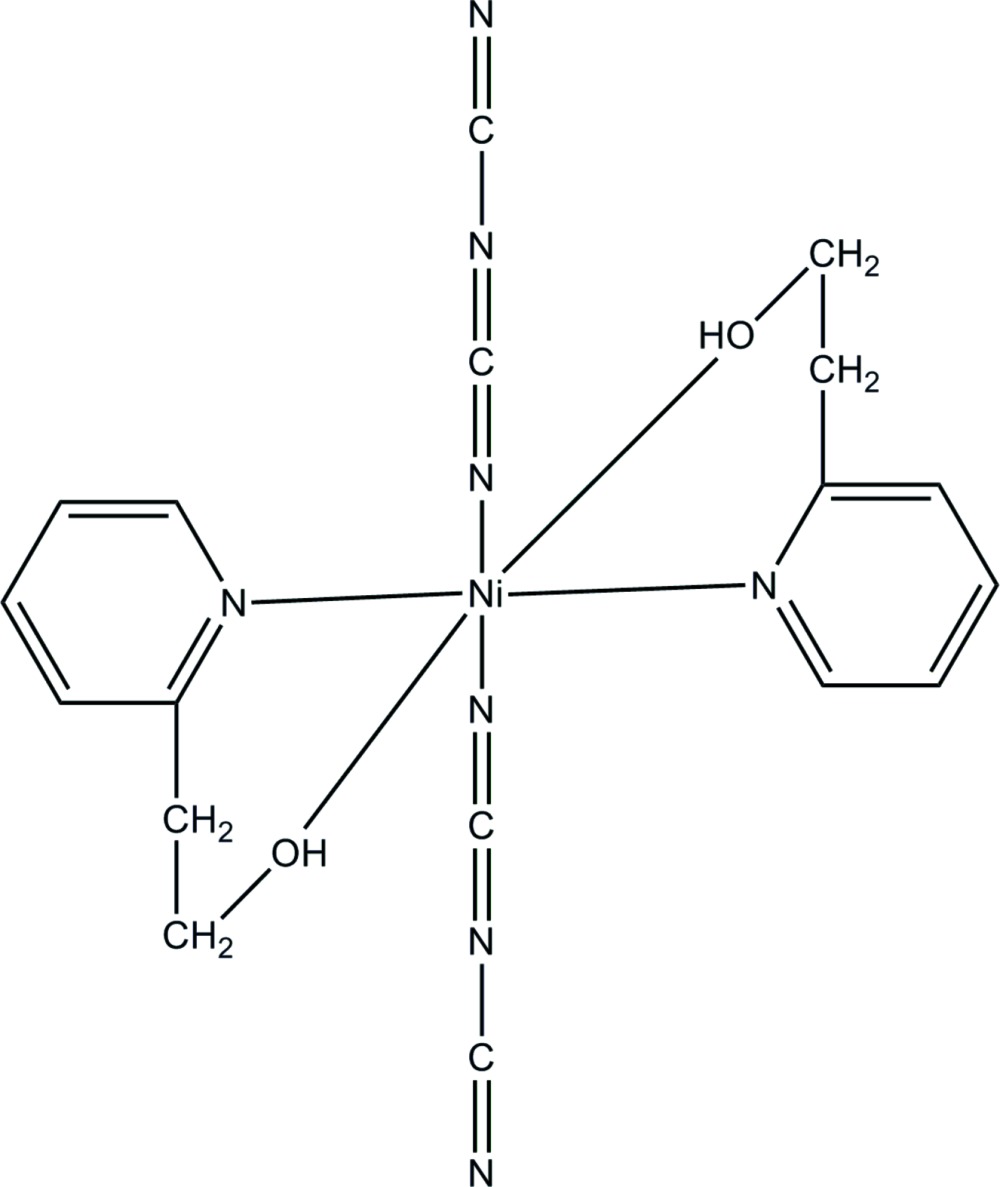


## Experimental

### 

#### Crystal data

[Ni(C_2_N_3_)_2_(C_7_H_9_NO)_2_]
*M* *_r_* = 437.11
Triclinic, 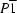

*a* = 8.1498 (1) Å
*b* = 8.76020 (11) Å
*c* = 8.9201 (12) Å
α = 100.841 (1)°
β = 110.588 (2)°
γ = 115.359 (2)°
*V* = 493.66 (7) Å^3^
*Z* = 1
Mo *K*α radiation
μ = 1.02 mm^−1^
*T* = 298 K
0.28 × 0.20 × 0.15 mm

#### Data collection

Bruker SMART CCD diffractometer
Absorption correction: multi-scan (*SADABS*; Bruker, 2003 ▶) *T*_min_ = 0.764, *T*_max_ = 0.863
2566 measured reflections
1718 independent reflections
1579 reflections with *I* > 2σ(*I*)
*R*_int_ = 0.016

#### Refinement

*R*[*F* ^2^ > 2σ(*F* ^2^)] = 0.034
*wR*(*F* ^2^) = 0.095
*S* = 1.00
1718 reflections
133 parameters
H-atom parameters constrained
Δρ_max_ = 0.53 e Å^−3^
Δρ_min_ = −0.22 e Å^−3^

### 

Data collection: *SMART* (Bruker, 2003 ▶); cell refinement: *SAINT* (Bruker, 2003 ▶); data reduction: *SAINT*; program(s) used to solve structure: *SHELXS97* (Sheldrick, 2008 ▶); program(s) used to refine structure: *SHELXL97* (Sheldrick, 2008 ▶); molecular graphics: *SHELXTL* (Sheldrick, 2008 ▶); software used to prepare material for publication: *SHELXTL*.

## Supplementary Material

Crystal structure: contains datablocks I, global. DOI: 10.1107/S1600536809043359/hb5097sup1.cif

Structure factors: contains datablocks I. DOI: 10.1107/S1600536809043359/hb5097Isup2.hkl

Additional supplementary materials:  crystallographic information; 3D view; checkCIF report

## Figures and Tables

**Table 1:** Selected bond lengths (Å)

Ni1—N2	2.065 (2)
Ni1—O1	2.0748 (16)
Ni1—N1	2.095 (2)

**Table 2:** Hydrogen-bond geometry (Å, °)

*D*—H⋯*A*	*D*—H	H⋯*A*	*D*⋯*A*	*D*—H⋯*A*
O1—H1⋯N4^i^	0.82	1.89	2.711 (3)	175

Symmetry code: (i) 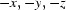.

**Fig. 1.**
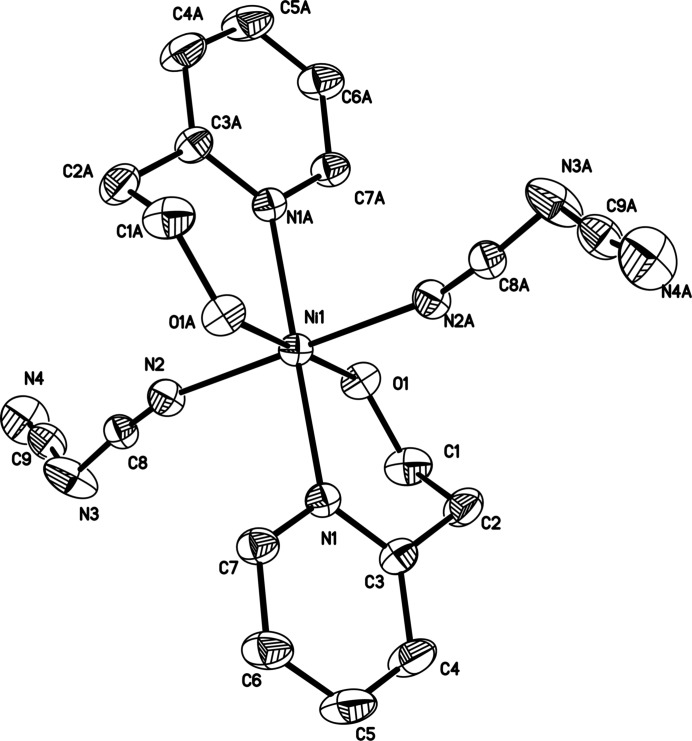
The structure of the title complex, showing 30% probability displacement ellipsoids. Atoms labelled with the suffix A are generated by the symmetry operation (-x + 1,-y,-z + 1). H atoms have been omitted for clarity.

**Fig. 2.**
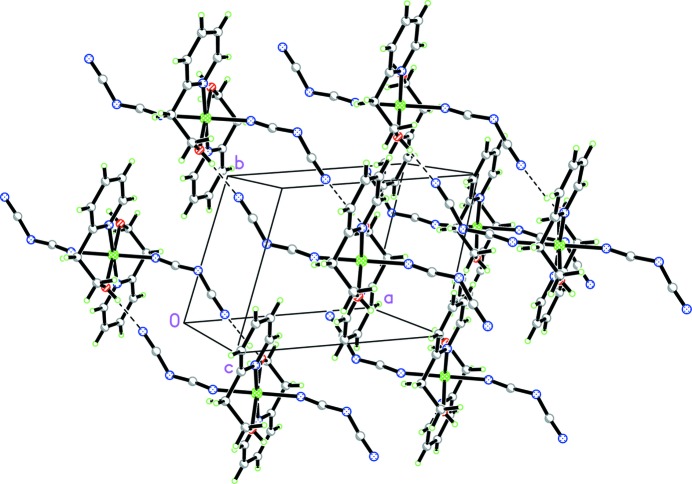
The crystal packing of (I), viewed approximately along the c axis.

## supplementary crystallographic information

### Comment

In recent years there has been considerable interest in metal complexes supported by hydroxyethyl-pyridine,the ligand due to its versatile coordination activities and bridging function. (Sanudo *et al.*, 2003; Boskovic *et al.*, 2002). As an extension of this work, we have synthesized the title compound, (I), and report herein its crystal structure.

The complex (Fig. 1) consists of two L2-(*L* = (hydroxyethyl)(pyridine)) ligands, one Ni^II^ ion and two dicyanmiden ligands. The coordination geometry around the Ni center is octahedral with a NiN_4_O_2_ ligand set (Table 1). Two atoms N1 of hydroxyethylpyridine ligand occpy the axial sites. In the crystal structure, intermolecular O—H···N hydrogen bonds link molecules to form a one-dimensional chain along to the *c* axis (Table 2).

### Experimental

2-Hydroxyethylpyridine (0.123 g, 1 mmol) was deprotonated by Et_4_NOH (25%) in the prensence of nickel nitrate hexahydrate (0.5 mmol, 0.127 g) in a mixture of methanol and acetonitrile (V/V = 1:1) after the solution was stirred at room temperature for 0.5 h. Sodium dicyanmiden (5 mmol 0.486 g) was added to the above solution and then further stirred for 1 h. The resulting clear solution was filtered and left to stand at room temperature. Green blocks of (I) were obtained by slow evaporation of the solvents within 2 weeks. MP = 518-520 K (decomp).

### Refinement

All H atoms were placed geometrically and treated as riding on their parent atoms with C—H = 0.93–0.97Å [*U*_iso_(H) = 1.2*U*_eq_(C)] and O—H = 0.82 Å [*U*_iso_(H) = 1.5*U*_eq_(O)].

### Crystal data

**Table:**

[Ni(C_2_N_3_)_2_(C_7_H_9_NO)_2_]	*Z* = 1
*M**_r_* = 437.11	*F*(000) = 226
Triclinic, *P*1	*D*_x_ = 1.47 Mg m^−^^3^
Hall symbol: -P 1	Mo *K*α radiation, λ = 0.71073 Å
*a* = 8.1498 (1) Å	Cell parameters from 1464 reflections
*b* = 8.76020 (11) Å	θ = 2.7–26.3°
*c* = 8.9201 (12) Å	µ = 1.01 mm^−^^1^
α = 100.841 (1)°	*T* = 298 K
β = 110.588 (2)°	Block, green
γ = 115.359 (2)°	0.28 × 0.20 × 0.15 mm
*V* = 493.66 (7) Å^3^	

### Data collection

**Table:**

Bruker SMART CCD diffractometer	1718 independent reflections
Radiation source: fine-focus sealed tube	1579 reflections with *I* > 2σ(*I*)
graphite	*R*_int_ = 0.016
ω scans	θ_max_ = 25.0°, θ_min_ = 2.7°
Absorption correction: multi-scan (*SADABS*; Bruker, 2003)	*h* = −9→9
*T*_min_ = 0.764, *T*_max_ = 0.863	*k* = −7→10
2566 measured reflections	*l* = −10→8

### Refinement

**Table:**

Refinement on *F*^2^	Primary atom site location: structure-invariant direct methods
Least-squares matrix: full	Secondary atom site location: difference Fourier map
*R*[*F*^2^ > 2σ(*F*^2^)] = 0.034	Hydrogen site location: inferred from neighbouring sites
*wR*(*F*^2^) = 0.095	H-atom parameters constrained
*S* = 1.00	*w* = 1/[σ^2^(*F*_o_^2^) + (0.0648*P*)^2^ + 0.0746*P*] where *P* = (*F*_o_^2^ + 2*F*_c_^2^)/3
1718 reflections	(Δ/σ)_max_ < 0.001
133 parameters	Δρ_max_ = 0.53 e Å^−^^3^
0 restraints	Δρ_min_ = −0.22 e Å^−^^3^

### Special details

**Table:**

Geometry. All e.s.d.'s (except the e.s.d. in the dihedral angle between two l.s. planes) are estimated using the full covariance matrix. The cell e.s.d.'s are taken into account individually in the estimation of e.s.d.'s in distances, angles and torsion angles; correlations between e.s.d.'s in cell parameters are only used when they are defined by crystal symmetry. An approximate (isotropic) treatment of cell e.s.d.'s is used for estimating e.s.d.'s involving l.s. planes.
Refinement. Refinement of *F*^2^ against ALL reflections. The weighted *R*-factor *wR* and goodness of fit *S* are based on *F*^2^, conventional *R*-factors *R* are based on *F*, with *F* set to zero for negative *F*^2^. The threshold expression of *F*^2^ > σ(*F*^2^) is used only for calculating *R*-factors(gt) *etc*. and is not relevant to the choice of reflections for refinement. *R*-factors based on *F*^2^ are statistically about twice as large as those based on *F*, and *R*- factors based on ALL data will be even larger.

### Fractional atomic coordinates and isotropic or equivalent isotropic displacement parameters (Å^2^)

**Table:**

	*x*	*y*	*z*	*U*_iso_*/*U*_eq_	
Ni1	0.5000	0.0000	0.5000	0.03081 (18)	
N1	0.7472 (3)	0.2759 (3)	0.6426 (3)	0.0341 (5)	
N2	0.4639 (3)	0.0453 (3)	0.2748 (3)	0.0415 (5)	
N3	0.3758 (4)	0.1688 (4)	0.0579 (4)	0.0700 (9)	
N4	0.0438 (5)	0.0438 (4)	−0.1949 (4)	0.0731 (9)	
O1	0.2970 (3)	0.0824 (2)	0.5031 (2)	0.0411 (4)	
H1	0.1971	0.0414	0.4067	0.062*	
C1	0.3611 (5)	0.2644 (4)	0.6012 (4)	0.0548 (8)	
H1A	0.2523	0.2612	0.6245	0.066*	
H1B	0.3849	0.3390	0.5343	0.066*	
C2	0.5577 (5)	0.3491 (4)	0.7713 (4)	0.0537 (8)	
H2A	0.5771	0.4562	0.8495	0.064*	
H2B	0.5422	0.2608	0.8249	0.064*	
C3	0.7468 (4)	0.4061 (4)	0.7512 (3)	0.0418 (6)	
C4	0.9168 (5)	0.5852 (4)	0.8400 (4)	0.0614 (9)	
H4	0.9143	0.6738	0.9136	0.074*	
C5	1.0883 (5)	0.6328 (4)	0.8202 (5)	0.0653 (9)	
H5	1.2039	0.7528	0.8817	0.078*	
C6	1.0887 (5)	0.5030 (4)	0.7096 (4)	0.0559 (8)	
H6	1.2034	0.5326	0.6932	0.067*	
C7	0.9155 (4)	0.3274 (4)	0.6227 (4)	0.0428 (6)	
H7	0.9151	0.2392	0.5456	0.051*	
C8	0.4109 (4)	0.0950 (4)	0.1684 (3)	0.0375 (6)	
C9	0.1941 (5)	0.0937 (4)	−0.0759 (4)	0.0489 (7)	

### Atomic displacement parameters (Å^2^)

**Table:**

	*U*^11^	*U*^22^	*U*^33^	*U*^12^	*U*^13^	*U*^23^
Ni1	0.0250 (3)	0.0319 (3)	0.0300 (3)	0.0149 (2)	0.00897 (19)	0.01179 (19)
N1	0.0305 (11)	0.0347 (11)	0.0322 (10)	0.0171 (9)	0.0116 (9)	0.0121 (9)
N2	0.0354 (12)	0.0455 (13)	0.0324 (11)	0.0182 (11)	0.0097 (10)	0.0168 (10)
N3	0.0383 (14)	0.0750 (19)	0.0627 (17)	0.0120 (13)	0.0049 (13)	0.0479 (16)
N4	0.0540 (17)	0.096 (2)	0.0586 (17)	0.0432 (17)	0.0088 (15)	0.0389 (17)
O1	0.0317 (9)	0.0441 (10)	0.0426 (10)	0.0243 (8)	0.0104 (8)	0.0135 (8)
C1	0.0432 (16)	0.0479 (17)	0.079 (2)	0.0307 (14)	0.0288 (16)	0.0222 (16)
C2	0.0537 (18)	0.0443 (16)	0.0553 (18)	0.0233 (15)	0.0298 (15)	0.0066 (14)
C3	0.0383 (14)	0.0379 (14)	0.0383 (14)	0.0186 (12)	0.0138 (12)	0.0090 (12)
C4	0.055 (2)	0.0382 (16)	0.065 (2)	0.0180 (15)	0.0240 (17)	0.0000 (15)
C5	0.0440 (18)	0.0349 (16)	0.084 (2)	0.0086 (14)	0.0234 (17)	0.0074 (16)
C6	0.0350 (15)	0.0443 (16)	0.072 (2)	0.0136 (13)	0.0231 (15)	0.0168 (15)
C7	0.0343 (14)	0.0380 (14)	0.0502 (16)	0.0177 (12)	0.0188 (13)	0.0140 (13)
C8	0.0251 (12)	0.0406 (14)	0.0343 (13)	0.0117 (11)	0.0108 (11)	0.0133 (12)
C9	0.0486 (17)	0.0551 (17)	0.0520 (17)	0.0306 (15)	0.0239 (15)	0.0326 (15)

### Geometric parameters (Å, °)

**Table:**

Ni1—N2^i^	2.065 (2)	C1—C2	1.506 (4)
Ni1—N2	2.065 (2)	C1—H1A	0.9700
Ni1—O1	2.0748 (16)	C1—H1B	0.9700
Ni1—O1^i^	2.0748 (16)	C2—C3	1.491 (4)
Ni1—N1	2.095 (2)	C2—H2A	0.9700
Ni1—N1^i^	2.095 (2)	C2—H2B	0.9700
N1—C7	1.337 (3)	C3—C4	1.381 (4)
N1—C3	1.353 (3)	C4—C5	1.365 (5)
N2—C8	1.142 (3)	C4—H4	0.9300
N3—C8	1.293 (3)	C5—C6	1.360 (5)
N3—C9	1.296 (4)	C5—H5	0.9300
N4—C9	1.127 (4)	C6—C7	1.372 (4)
O1—C1	1.422 (3)	C6—H6	0.9300
O1—H1	0.8200	C7—H7	0.9300
			
N2^i^—Ni1—N2	180.0	O1—C1—H1B	109.6
N2^i^—Ni1—O1	92.61 (8)	C2—C1—H1B	109.6
N2—Ni1—O1	87.39 (8)	H1A—C1—H1B	108.1
N2^i^—Ni1—O1^i^	87.39 (8)	C3—C2—C1	113.5 (3)
N2—Ni1—O1^i^	92.61 (8)	C3—C2—H2A	108.9
O1—Ni1—O1^i^	180.0	C1—C2—H2A	108.9
N2^i^—Ni1—N1	91.92 (8)	C3—C2—H2B	108.9
N2—Ni1—N1	88.08 (8)	C1—C2—H2B	108.9
O1—Ni1—N1	89.07 (7)	H2A—C2—H2B	107.7
O1^i^—Ni1—N1	90.93 (7)	N1—C3—C4	120.6 (3)
N2^i^—Ni1—N1^i^	88.08 (8)	N1—C3—C2	117.7 (2)
N2—Ni1—N1^i^	91.92 (8)	C4—C3—C2	121.7 (3)
O1—Ni1—N1^i^	90.93 (7)	C5—C4—C3	120.3 (3)
O1^i^—Ni1—N1^i^	89.07 (7)	C5—C4—H4	119.8
N1—Ni1—N1^i^	180.0	C3—C4—H4	119.8
C7—N1—C3	117.8 (2)	C6—C5—C4	119.4 (3)
C7—N1—Ni1	117.74 (17)	C6—C5—H5	120.3
C3—N1—Ni1	124.45 (18)	C4—C5—H5	120.3
C8—N2—Ni1	156.7 (2)	C5—C6—C7	118.3 (3)
C8—N3—C9	122.3 (3)	C5—C6—H6	120.8
C1—O1—Ni1	124.15 (16)	C7—C6—H6	120.8
C1—O1—H1	109.5	N1—C7—C6	123.6 (3)
Ni1—O1—H1	113.9	N1—C7—H7	118.2
O1—C1—C2	110.2 (2)	C6—C7—H7	118.2
O1—C1—H1A	109.6	N2—C8—N3	172.5 (3)
C2—C1—H1A	109.6	N4—C9—N3	173.2 (3)
			
N2^i^—Ni1—N1—C7	115.4 (2)	O1—C1—C2—C3	74.7 (3)
N2—Ni1—N1—C7	−64.6 (2)	C7—N1—C3—C4	−1.0 (4)
O1—Ni1—N1—C7	−152.00 (19)	Ni1—N1—C3—C4	−179.8 (2)
O1^i^—Ni1—N1—C7	28.00 (19)	C7—N1—C3—C2	179.1 (3)
N2^i^—Ni1—N1—C3	−65.8 (2)	Ni1—N1—C3—C2	0.3 (3)
N2—Ni1—N1—C3	114.2 (2)	C1—C2—C3—N1	−56.9 (4)
O1—Ni1—N1—C3	26.8 (2)	C1—C2—C3—C4	123.2 (3)
O1^i^—Ni1—N1—C3	−153.2 (2)	N1—C3—C4—C5	−0.5 (5)
O1—Ni1—N2—C8	9.2 (5)	C2—C3—C4—C5	179.4 (3)
O1^i^—Ni1—N2—C8	−170.8 (5)	C3—C4—C5—C6	1.3 (6)
N1—Ni1—N2—C8	−80.0 (5)	C4—C5—C6—C7	−0.6 (5)
N1^i^—Ni1—N2—C8	100.0 (5)	C3—N1—C7—C6	1.8 (4)
N2^i^—Ni1—O1—C1	84.2 (2)	Ni1—N1—C7—C6	−179.3 (2)
N2—Ni1—O1—C1	−95.8 (2)	C5—C6—C7—N1	−1.0 (5)
N1—Ni1—O1—C1	−7.7 (2)	Ni1—N2—C8—N3	104 (2)
N1^i^—Ni1—O1—C1	172.3 (2)	C9—N3—C8—N2	177 (2)
Ni1—O1—C1—C2	−34.9 (3)	C8—N3—C9—N4	172 (3)

Symmetry codes: (i) −*x*+1, −*y*, −*z*+1.

### Hydrogen-bond geometry (Å, °)

**Table:**

*D*—H···*A*	*D*—H	H···*A*	*D*···*A*	*D*—H···*A*
O1—H1···N4^ii^	0.82	1.89	2.711 (3)	175

Symmetry codes: (ii) −*x*, −*y*, −*z*.
